# Toxicity, Bioaccumulation and Biotransformation of Glucose-Capped Silver Nanoparticles in Green Microalgae *Chlorella vulgaris*

**DOI:** 10.3390/nano10071377

**Published:** 2020-07-15

**Authors:** Stefania Mariano, Elisa Panzarini, Maria D. Inverno, Nick Voulvoulis, Luciana Dini

**Affiliations:** 1 Department of Biological and Environmental Science and Technology, University of Salento, 73100 Lecce, Italy; stefania.mariano@unisalento.it (S.M.); elisa.panzarini@unisalento.it (E.P.); 2 Centre for Environmental Policy, Imperial College London, London SW7 2AZ, UK; mjoaoinverno@gmail.com (M.D.I.); n.voulvoulis@imperial.ac.uk (N.V.); 3 Department of Biology and Biotechnology “Charles Darwin”, Sapienza University of Rome, 00185 Rome, Italy; 4 CNR Nanotec, 73100 Lecce, Italy

**Keywords:** *Chlorella vulgaris*, silver nanoparticles, ecotoxicity, growth inhibition, chlorophyll-a content, morphological changes, bioaccumulation, crystalline structure

## Abstract

Silver nanoparticles (AgNPs) are one of the most widely used nanomaterials in consumer products. When discharged into the aquatic environment AgNPs can cause toxicity to aquatic biota, through mechanisms that are still under debate, thus rendering the nanoparticles (NPs) effects evaluation a necessary step. Different aquatic organism models, i.e., microalgae, mussels, *Daphnia magna*, sea urchins and *Danio rerio*, etc. have been largely exploited for NPs toxicity assessment. On the other hand, alternative biological microorganisms abundantly present in nature, i.e., microalgae, are nowadays exploited as a potential sink for removal of toxic substances from the environment. Indeed, the green microalgae *Chlorella vulgaris* is one of the most used microorganisms for waste treatment. With the aim to verify the possible involvement of *C. vulgaris* not only as a model microorganism of NPs toxicity but also for the protection toward NPs pollution, we used these microalgae to measure the AgNPs biotoxicity and bioaccumulation. In particular, to exclude any toxicity derived by Ag^+^ ions release, green chemistry-synthesised and glucose-coated AgNPs (AgNPs-G) were used. *C. vulgaris* actively internalised AgNPs-G whose amount increases in a time- and dose-dependent manner. The internalised NPs, found inside large vacuoles, were not released back into the medium, even after 1 week, and did not undergo biotransformation since AgNPs-G maintained their crystalline nature. Biotoxicity of AgNPs-G causes an exposure time and AgNPs-G dose-dependent growth reduction and a decrease in chlorophyll-a amount. These results confirm *C. vulgaris* as a bioaccumulating microalgae for possible use in environmental protection.

## 1. Introduction

In the last few decades, nanoparticles (NPs) have attracted great attention due to their chemical, physical, optic and biological properties. Accordingly, safety assessment becomes an important issue for the beneficial usage of these new materials [1]. NPs chemical and physical properties (chemical composition, size, shape) and the complex interactions occurring at various biological levels (organelle, cell, tissue, organ, organ system, organism) can potentially impact on human health [2]. Some studies have shown that NPs cause toxic effects which are instead not induced by similar but larger particles. These effects are probably due to the intrinsic characteristics of the NPs which permit targets that cannot be reached by their larger and chemically identical counterparts to be achieved [3]. The human body can interact with nanomaterials (NMs) mainly through different ways: inhalation through the respiratory system, ingestion through the gastrointestinal tract, and absorption through the skin. Once inside, they manage to overcome further barriers, such as the brain–blood barrier, causing toxic effects on human health [2]. Several studies demonstrated toxic effects of NPs on human health, including those associated with cardiovascular disease derived from titanium dioxide, metal oxide and metal nanoparticles exposure [4], and pulmonary inflammation induced by carbon containing NMs [5,6]. These effects include among others inflammation, granuloma formation, and fibrosis of the lungs [5,7].

Together with the increasing nanobiotechnological application, exposure to NPs of all living organisms and the environment enhances and justifies the need to identify, measure and manage the risks.

The environmental fate and transport models demonstrated that NPs/NMs can enter, as nanowaste, directly or indirectly, soil and waterways [8]. Thus, through washing, rain and other routes, these NPs/NMs can be released especially into the aquatic environment, where they can be potentially toxic to biota causing an ecological impact as well as to humans with socioeconomic consequences [9,10,11].

Silver nanoparticles (AgNPs) are frequently used in consumer products or medical devices for their antibacterial and antifungal activities [12,13], being the most studied in the field of nanoecotoxicology. Their widespread use in commercial products, mainly because of their bacterial power, has led to a steadily increasing amount of AgNPs in environment [14]. Most of the environmental concerns are raising the fate of AgNPs in washing machines, textile industry and similar applications. The release of AgNPs and Ag^+^ into the water by simply immersing commercial socks containing AgNPs into shaken water was revealed by Benn et al. [15]. Consumables containing AgNPs have been subjected to different treatments, such as interaction with surfactants, oxidising agents, different pH, to test the release of AgNPs during the washing processes and the passage of these NPs in the sweat of human skin. These treatments greatly increased the release of Ag^+^ and AgNPs [16,17] and became an important route for increasing the NPs in the environment.

In addition, industrial treatment of NPs can lead to the release of AgNPs into the sewage system or wastewater [18]. Shafer et al. [19] measured total silver concentrations (non-nanospecific) of up to 105 µg/L in the liquid inflow of a wastewater treatment plant. It has been proven that many of the AgNPs is retained in the sewage sludge during the wastewater treatment process. However, a smaller part of the AgNPs can still reach the environment via the effluent [20].

Against this background, a wide variety of organisms, i.e., bacteria, plants, fungi, algae, invertebrates and fish have been considered to evaluate the behavior of AgNPs with aquatic organisms.

Knowledge gaps on how AgNPs interact with a living-organisms remain an issue at all levels of organisation, in particular at a cellular and molecular level (genes, transcripts, metabolites, proteins, enzymes and soluble factors). However, the effects of AgNPs on aquatic algal microorganisms was reported to induce time and concentration changes in speciation of microalgae *Raphidocelis subcapita* [21]; inhibit growth and cellular viability of the diatom *Thalassiosira pseudonana*, cyanobacterium *Synechococcus* sp. [22] but also of the aquatic plant *Lemna gibba* [23]; and favour superoxide production in the marine raphidophyte *Chattonella marina* [24]. Again, AgNPs affect the photosynthesis process, leading to a change in the chlorophyll content of algae *Chlamydomonas reinhardtii* [25] or cyanobacterium *Microcystis aeruginosa* [26], algae *Pithophora oedogonia* and algae *Chara vulgaris* [27], microalgae *Acutodesmus dimorphus* [28] and green algae *Chlamydomonas reinhardtii* [29].

Among the different strategies for reducing the NPs’ environmental impact and preventing the potentially toxic effects of AgNPs due to the release of Ag^+^ or to the agglomeration of particles in aqueous systems, green chemistry has been introduced in the synthesis of AgNPs and surface coating for stabilisation [30,31,32]. Taken together these two technological approaches that use innovative principles in the design of industrial chemical processes, could be fundamental for achieving sustainable industrial development, preventing and reducing industrial pollution and environmental impact. Indeed, green chemistry promotes the design, manufacture and use of chemicals and processes that abolish or reduce the use or generation of substances injurious to environment and health [33]. To this purpose, the use of natural sources, non-hazardous solvents, biodegradable and biocompatible materials, such as cellulose, chitosan, dextran or tree gums, and energy-efficient processes are the main NPs’ preparation innovation [34,35,36].

Considering that the zero release of AgNPs into water is not realistic, synergistic approaches to the technology of NPs synthesis should be considered, such as the use of microorganisms as bioaccumulators and/or biotransformators. In fact, during the last two decades, several methods have been developed for environmental removal of hazardous substances like precipitation, evaporation, ion-exchange etc., even if these methods have several disadvantages [37,38]. One alternative strategy is the use of microorganisms abundantly present in nature, i.e., microalgae, that are already used to remove heavy metals and in wastewater treatment facilities; in fact, the microalgae reduce the amount of toxic chemicals needed to clean and purify water [39], being able either to accumulate, adsorb or metabolise these noxious elements into a substantial level.

However, studies on the ability of microalgae to remedy NPs aquatic pollution are still very limited [40]. In this study, we used green chemistry-synthesised AgNPs, that were capped with glucose-G (AgNPs-G) to ensure AgNPs stability [41,42,43] and *Chlorella vulgaris*, one of the most widely used microorganism in testing NPs/NMs effects on aquatic biota but also known to reduce heavy metals from waters [37,38]. Among aquatic organisms, algae are an important model as they are primary producers, i.e., they fix CO_2_ to produce oxygen in the presence of light. Also, they are at the base of the food chain, serving as a food to, e.g., the water flea but also fish. The microalgae *C. vulgaris* was chosen in the present study because of its easy growth in commercial culture. This unicellular green species with a cell diameter of 5 µm, has been utilised for varied purposes, ranging from nutrient removal from wastewater to their use as a food source. Its ability to survive in adverse conditions and its ubiquitous nature also make it a potentially useful algae for industrial wastewater treatment. Specifically, the microalgae *Chlorella vulgaris* is known for its robustness, in conjunction with being one of the fastest growing species and is easily cultivated [44].

To exploit the ability of *C. vulgaris* in the removal of AgNPs-G from water, we investigated the efficiency of the microalgae to uptake and retain NPs. Studies of AgNPs-G characterisation and nanotoxicology were also performed.

## 2. Materials and Methods

### 2.1. Chemicals

All chemicals were of analytical grade and were purchased from Sigma-Aldrich (St. Louis, MO, USA) unless otherwise indicated.

### 2.2. Synthesis of Glucose-Capped Silver Nanoparticles (AgNPs-G)

AgNPs-G were obtained by adding 2 mL of a 10^−2^ M aqueous solution of AgNO_3_ to 100 mL of 0.3 M β-D-glucose water solution. The mixture was boiled for 30 min under vigorous stirring. The deep yellow colour of the solutions indicated the formation of AgNPs-G. Deionised ultra-filtered 18.2 MΩ water prepared with a Milli-Q Integral Water Purification System (Merck Millipore, Billerica, MA, USA) was used for all preparations. All glassware was washed in an ultrasonic bath of deionised water and not ionic detergent, followed by thorough rinsing with Milli-Q water and ethanol (Carlo Erba, Milan, Italy) to completely remove not ionic detergent contaminants. Finally, glassware was dried prior to use.

### 2.3. AgNPs-G Characterisation

Transmission electron microscopy (TEM) and ultraviolet–visible (UV–Vis) analysis were used to evaluate the average and distribution size and morphology of the NPs.

TEM analysis was performed by a Hitachi 7700, at 100 kV (Hitachi, Dallas, TX, USA). A drop of AgNPs-G solution diluted in complete Bold’s basal medium (BBM) [45] was placed onto standard carbon-supported 600-mesh copper grid. Particle size distribution has been obtained using the ImageJ program (National Institutes of Health (NIH), Bethesda, MD, USA). A histogram was created by counting 500 particles. Optical spectra were obtained by measuring the absorption of the solution in the range between 300 and 800 nm by using a T80 spectrophotometer (PG Instruments Ltd., Leicester, UK) in a quartz cuvette with a 1 cm optical path.

The stability of AgNPs-G was assayed in BBM. In particular, the dissolution of AgNPs-G, in terms of release of Ag^+^, up to 10 days at r.t. (room temperature) in BBM culture medium was determined by atomic absorption spectroscopy (AAS; Thermo Electron Corporation, M-Series, Waltham, MA, USA) after precipitation of AgNPs-G by ultracentrifugation (24,900× *g*; 30 min at 4 °C). The detection limit was 1 μg/L. Triplicate readings were analysed and control samples of known Ag concentration were analysed in parallel generating data with the standard deviation of three independent samples. Silver ions dissolution degree was expressed as percentage (%) of total Ag^+^, as AgNO_3_, used to reach the highest concentration of NPs solution during treatment.

Bold’s basal medium composition: NaNO_3_ 250 mg/L, K_2_HPO_4_ 75 mg/L, MgSO_4_.7H_2_O 75 mg/L, CaCl_2_.2H_2_O 25 mg/L, KH_2_PO_4_ 175 mg/L, NaCl 25 mg/L, Alkaline Ethylenediaminetetraacetic Acid (EDTA) solution 1 mg/L (alkaline EDTA solution: 5 g Na_2_-EDTA and 3.1 g KOH in 100 mL distilled water), acidified iron solution 1 mg/L (acidified iron solution FeSO_4_.7H_2_O 498 g and 0.1 mL H_2_SO_4_ in 100 mL distilled water) trace metal solution 1 mL/L (trace metal solution: MnCl_2_.4H_2_O 1.44 g/L, ZnSO_4_.7H_2_O 8.82 g/L, (NH_4_)_6_ Mo_7_O_24_.2H_2_O 0.88 g/L, Co(NO_3_)_2_.6H_2_O 0.49 g/L, CuSO_4_.5 H_2_O 1.57 g/L).

### 2.4. Chlorella Vulgaris Culture

The freshwater microalga *C. vulgaris* was obtained from the Culture Collection of Algae and Protozoa (Argyll, UK). The algae were cultured in 250 mL flasks containing 100 mL of BBM and covered with loose cotton. The flasks were placed on a shaker to keep the turbulence of culture medium simulating the natural stream of water. The cultures were kept at 23 ± 1 °C under illumination of approximately 73.6 μmol m^−2^ s^−1^ with daily cycles of 12 h light and 12 h dark. The culture cell density was monitored with a spectrophotometer (Pharmacia Biotech, Stockholm, Sweden) at 684 nm every 24 h. Cells in the exponential phase were used for all experiments.

### 2.5. Growth-Inhibition Test

The evaluation was performed following the Organisation for Economic Co-operation and Development (OECD) 201 algal growth inhibition test guidelines [46]. Algae were incubated for 24 h and a week with Ag ions (0.1 μg/L and 1 mg/L of silver nitrate) and with different concentrations of AgNPs-G: 0.1, 1, 10, 100 μg/L and 1 mg/L with three replicates for each concentration. The inhibitory rate of growth was obtained by using the Equation (1):

Inhibitory Rate (*IR*)% = (1 – *N*/*N*_0_) × 100 (1)

where *N* is the density of cells/mL in the samples treated with AgNPs-G, *N*_0_ is the density of cells/mL in the control samples. The test was performed with three independent experiments (with three technical replicates for each repeated experiment) by using the same batch of algae and AgNPs-G.

### 2.6. Chlorophyll Content

Treated samples were centrifuged to remove culture media. Then, 90% acetone was added to tubes. Sealed tubes were shaken to ensure that microalgae cells are in the whole solvent volume and centrifuged at 5000 rpm (5236× *g*) for 5 min. Chlorophyll-a concentration was determined by measuring the optical density (OD) of supernatant by spectrophotometer (Pharmacia Biotech, Stockholm, Sweden). Absorbance values of extracts were measured at 645 and 663 nm in 1 cm pathlength cuvettes. Quantitative determination was undertaken according to Arnon (1949) [47].

### 2.7. Biodistribution and Subcellular Localisation of AgNPs: Transmission Electron Microscope (TEM) Analysis

The ultrastructural analysis of *C. vulgaris* treated with different concentrations of Ag ions and AgNPs-G for one day and one week was performed by TEM (Hitachi HT 7700 transmission electron microscopy) analysis.

Algae were centrifuged to remove culture media and then fixed with glutaraldehyde (2.5% in sodium cacodilate buffer 0.1 M, pH 7.2) for 2 h at 4 °C. Then, samples were washed twice for 15 min in sodium cacodilate buffer, postfixed in osmium tetraoxide (1% in sodium cacodilate buffer 0.1 M, pH 7.2) and washed twice for 30 min in deionised H_2_O. Samples were stained with 0.5% uranyl acetate o.n. (over night) at 4 °C. Samples were dehydrated in a graded series of ethanol, from 30% to 100%. After dehydration, samples were embedded in Spurr resin (TAAB, Berks, UK).

Ultrathin sections of 50 nm in thickness were then cut using an ultramicrotome PowerTome PT-PC (RMC, Tucson, AZ, USA). Sections were picked up in 200 mesh copper grids and examined under a Hitachi HT7700 transmission electron microscope (Tokyo, Japan) at 75 kV.

Samples were analyzed by energy-dispersive X-ray spectroscopy (EDX) microanalysis with the TEM module of the Auriga 405 microscope (Carl Zeiss AG, Oberkochen, Germany) for the elemental analysis of the electron-dense particles inside the cells.

### 2.8. X-ray Diffraction (XRD) Analysis

To determine the amount of Ag^+^ inside algal cells, X-ray diffraction (XRD) analysis was performed with samples of algae treated for a week with AgNPs-G. Only AgNPs-G were used as positive control and a culture of only *C. vulgaris* as negative control. Samples were collected, dried at 60 °C and then sintered at 650 °C for 4h under nitrogen protection. The analysis was performed with X-ray diffractometers (Malvern Pananalytical, Malvern, UK).

### 2.9. Inductively Coupled Plasma–Optical Emission Spectrometry (ICP–OES) Analysis

A series of AgNPs-G stocks (0.1, 1, 10, 100 μg/L and 1 mg/L) were prepared in BBM. Algal samples with different AgNPs-G exposure concentrations and times were vacuum filtered with a 0.45 μm Millipore filter to separate algae from the culture medium. Samples were acidified with HNO_3_ and analysed by inductively coupled plasma–optical emission spectroscopy (ICP–OES, Perkin Elmer Optima 7300 V HF version, Waltham, MA, USA) to determine Ag content. ICP–OES is a technique commonly used for the analysis of metals in various fields based on Atomic Emission Spectroscopy, where the sample at high temperature plasma up to 8000 K is converted to free, excited or ionised ions. The ions emit a radiation when go back to ground state, whose intensities are optically measured and indicate the amount of ions. The absorbed Ag by algal cells was calculated by the total Ag (*T*_Ag_, also determined by ICP–OES by measuring stock solutions) minus the Ag in filtrates (*F*_Ag_). Therefore, the percentage of absorbed Ag was calculated as (*T*_Ag_ − *F*_Ag_)/*T*_Ag_ × 100.

### 2.10. Statistical Analysis

Data were analysed by performing one-way analysis of variance (ANOVA) at the 95% confidence level. *p* values less than 0.05 were considered significant. The results are reported as mean ± standard deviation (SD) of 3 technical replicates in each of the 3 independent experiments.

## 3. Results and Discussion

### 3.1. Characterisation of AgNPs-G: Shape, Size and Stability

**Figure 1:**
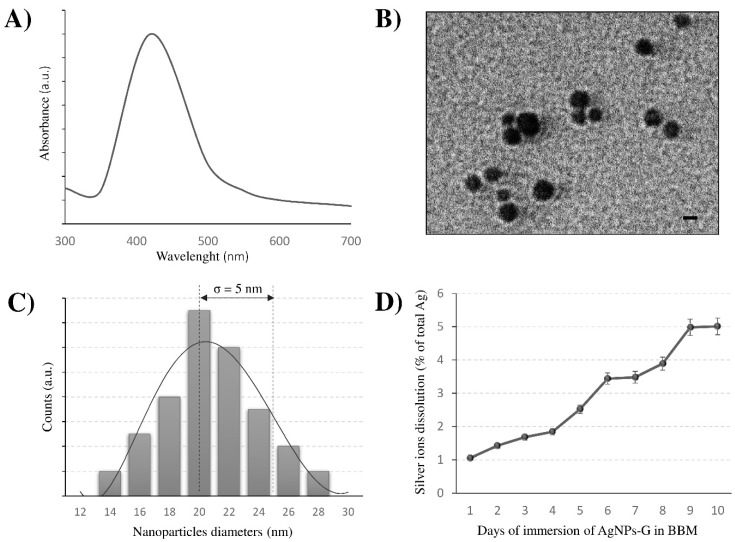
(**A**) Ultraviolet (UV)–visible spectra of glucose-capped silver nanoparticles (AgNPs-G)/mL in Bold’s basal medium (BBM) culture medium reported as absorbance in arbitrary unit (a.u., y axis) vs. wavelength (nm, x axis). (**B**,**C**) Size distribution and transmission electron microscopy (TEM) micrograph of AgNPs-G. Size distribution is reported as arbitrary unit (a.u., y axis) vs. longitudinal diameter (nm, x axis). Bars = 20 nm. (**D**) Kinetic of Ag^+^ dissolution. The dissolution of AgNPs-G in complete BBM culture medium was evaluated by atomic absorption spectroscopy. Data were analysed by performing one-way analysis of variance (ANOVA) at the 95% confidence level. Each value represents the mean ± standard deviation (SD) of 3 technical replicates in each of the 3 independent experiments. Ag^+^ dissolution degree is expressed as percentage (%) of total AgNO_3_ used to obtain the highest concentration of NPs solution during treatment.

Uptake and/or toxic effects rely on the shape, size and dispersion of the NPs. AgNPs-G shape, average size and size distribution, evaluated by TEM and UV–visible spectra, are reported in Figure 1. The AgNPs-G UV–visible absorbance spectrum (Figure 1A) shows a characteristic absorption wavelength of spheroidal AgNPs, as suggested by a strong extinction band with a maximum at 420 nm. TEM showed spherical shape and good monodispersity of AgNPs-G (Figure 1B). The size distribution ranges from 14 to 28 nm and the average size is *d* = 20 nm with a standard deviation of 5 nm (Figure 1C).

It is known that particle toxicity could depend on Ag^+^ released from NPs, thus the stability of AgNPs in the culture medium was estimated up to 10 days, in terms of Ag^+^ release, by atomic absorption spectroscopy (Figure 1D). AgNPs-G were very stable in culture medium over time, since the dissolution degree, expressed as a percentage of total Ag^+^ ranges between 1% and 5% at 1 and 10 days respectively. Since β-D-glucose capping ensures very low dissolution of Ag^+^ from AgNPs and no loss of glucose was observed, the toxicity is due only to NPs.

In aquatic environments, dissolved oxygen in water oxidises the AgNPs surface causing Ag^+^ ions release [48], identified as one of the most phytotoxic metal ions [49] for their cationic property and for the ability to associate with a variety of ligands present in natural waters. The toxicity of NP-released Ag^+^ ions was reported for the alga *Chlamydomonas reinhardtii* [50,51], while Turner et al. [52] reported that AgNPs are only indirectly toxic to marine algae *Ulva lactuca* through the dissolution of Ag^+^ ions into bulk seawater. However, whether AgNPs toxicity is due to the nanosized structure or to the released silver ions is still a matter of debate, and the results seem to be contingent mainly on the features of the AgNPs considered.

In our experiments, biotoxicity is not due to the Ag^+^ ions release or the nanoparticle aggregates. To reduce as much as possible the Ag^+^ release we used β-D-glucose for the green chemistry synthesis of AgNPs on the base of our previous data indicating that AgNPs-G are stable, well-dispersed with a minimum Ag^+^ release in culture medium [42]. Indeed, in our experiments we measured the release of only 5% of the amount of the NPs after 10 days in seawater, thus confirming the effectiveness of the synthesis based on β-D-glucose as a reducing agent. The reduction of NPs toxicity by surface functionalisation with different coatings was also observed in several other studies [53,54]. Possible explanations could be attributed to the reduced nanoparticle dissolution as well as to the limited interactions between nanoparticles and organisms. For example, dexpanthenol, polyethylene glycol and polyvinyl polypyrrolidone coatings caused a similar toxic effect as AgNO_3_ on *C. reinhardtii*, while carbonate, chitosan and citrate decreased the Ag effect on photosynthesis [29]. *C. vulgaris* were exposed to Ag^+^ ions to understand if AgNP-G toxicity is driven by dissolved silver. The highest concentration of Ag^+^ given as AgNO_3_, was 100 times more the estimated release of AgNPs to the aquatic environment, that is about 0.01 mg/L^−1^ [55] and undoubtedly underestimated since this amount will increase in the near future for the forecast usage of these nanoparticles [56]. Our data showed that Ag^+^ ions have only minimal effects on cell growth, morphological alteration, chlorophyll-a content and that the high doses of AgNPs-G only significantly reduced these parameters.

Toxicity of AgNPs has been a controversial topic for a long time. The open question is still the understanding of the toxicity mechanism of AgNPs. It seems not to be limited to the Ag^+^ ions release but to different factors including the nanostructure [1]. According to Domingo et al. [57], AgNPs toxicity is not fully attributable to released ions since in photosynthetic organisms Ag^+^ ions and AgNPs caused similar effects, although Ag^+^ ions were often active at lower concentrations. Possible transformations of AgNPs-G mainly due to the aquatic chemistry cannot be excluded. Data in literature show that the aggregation of NPs in water depends on different parameters such as the pH or the surface charge of the NPs involved and by the specific type of organic matter or other natural particles present in freshwater [58]. In addition, AgNPs toxicity may depend on the species and on the type of growth medium in which the organisms are cultivated [59]. However, some adverse effects can also be attributed to specific properties of NPs, such as the size and the degree of aggregation, that in seawater is increased when compared to freshwater [60,61], and that in turn affect the capacity of NPs to cross biological membranes or bind the cell surface [62,63]. Cell wall, in fact, constitutes a primary site for interaction and serves as a barrier for the entrance of AgNPs into algal cells. In our hands, even after 1 week from the synthesis, AgNPs-G diluted in BBM were stable and well dispersed.

### 3.2. AgNPs-G are Bio-Absorbed by *C. vulgaris* Maintaining Their Crystalline Structure

**Figure 2:**
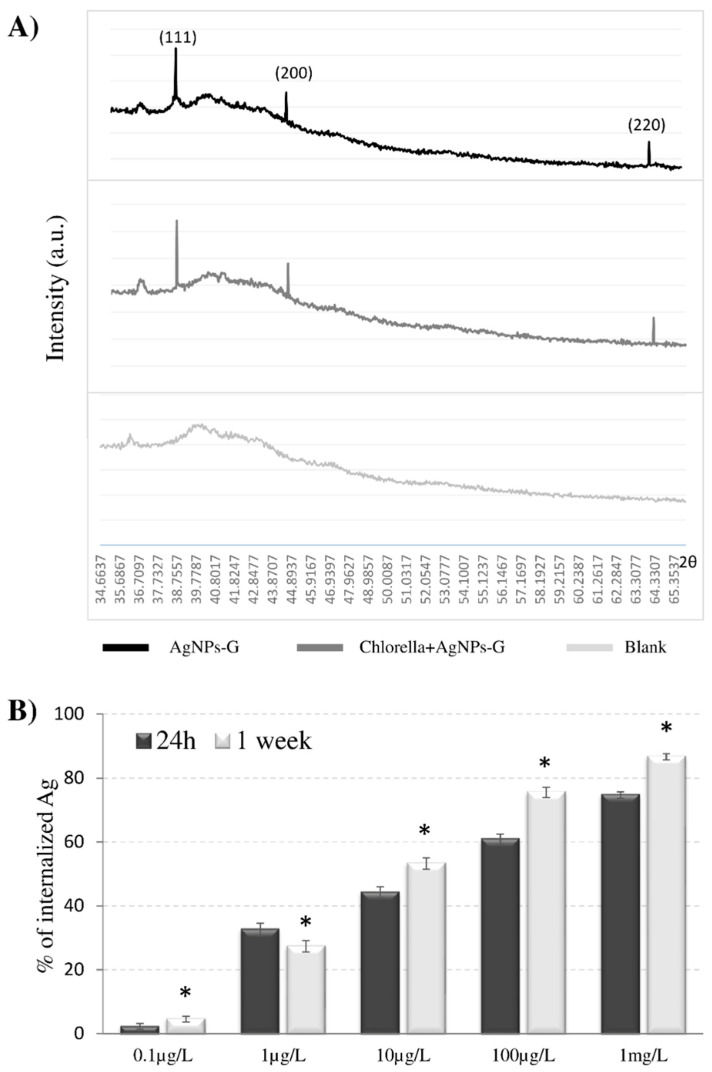
(**A**) X-ray diffraction (XRD) spectrum of AgNPs-G before and after the interaction with algae. A culture of *C. vulgaris* is used as negative control. Numbers refer to diffraction peaks of Ag in its crystalline form. (**B**) Inductively coupled plasma–optical emission spectrometry (ICP–OES) to determine Ag internalisation by algal cells treated with five concentrations of AgNPs-G. The absorbed Ag was calculated by the total Ag (*T*_Ag_, also determined by ICP–OES by using stocks at five concentrations) minus the Ag in filtrates (*F*_Ag_). Therefore, the percentage of absorbed Ag = (*T*_Ag_ − *F*_Ag_)/*T*_Ag_ × 100. Data were analysed by performing one-way ANOVA at the 95% confidence level. Each value represents the mean ± SD of 3 technical replicates in each of the 3 independent experiments. Asterisks indicate significant differences from respective values at 24 h at the same concentration (*p* < 0.05).

**Figure 3:**
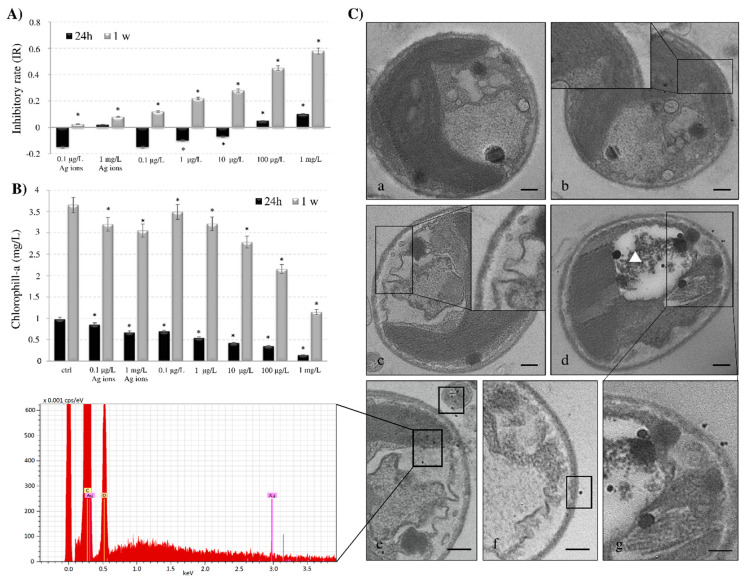
(**A**) Analysis of inhibitory rate. Algae were incubated for 24 h and a week with Ag ions and with five concentrations of AgNPs-G. Data were analysed by performing one-way ANOVA at the 95% confidence level. Each value represents the mean ± SD of 3 technical replicates in each of the 3 independent experiments. Asterisks indicate significant differences from the control values (*p* < 0.05); (**B**) Analysis of chlorophyll-a content by spectrophotometric analysis of centrifuged samples. Quantitative determination was done according to Arnon et al. (1949). The experiments were conducted in triplicate and results are the mean with standard deviation. Asterisks indicate significant differences from the respective untreated samples (*p* < 0.05) (**C**) TEM micrographs of algal cells and elemental X-ray spectrum (lower panel) of the square area of micrograph (**e**) containing black spots. (**a**) control cell; (**b**) algal cells treated with Ag ions. (**c**) algal cells treated with AgNPs-G for 24 h. Plasma membrane detaches from the cell wall, as indicated in the magnification; (**d**–**g**) Algal cells treated with AgNPs-G for a week. AgNPs-G were observed inside large vacuoles (**d**, white triangle), inside algae (**d**–**e**) or crossing the cell wall (**f**–**g**). Bar = 500 nm.

*C. vulgaris*, at the exponential growth phase, was exposed to Ag^+^ (0.1 μg/L and 1 mg/L of silver nitrate) or to different AgNPs-G concentrations (0.1, 1, 10, 100 μg/L and 1 mg/L) for 1 day or 1 week. In order to ensure that the cytotoxic effect (in terms of cell viability, chlorophyll content and ultrastructural changes) of silver nanoparticles is not due to the presence of Ag^+^ ions in the suspension, AgNO_3_, the salt of which the nanoparticles are made, has been used to prepare two solutions with a range that covers the amounts of Ag released in 10 days (from 1% to 5%) in the nanoparticles stability analysis. Moreover, in order to test our glucose-capped AgNPs on *C. vulgaris*, we chose scalar dilutions of NPs following data in literature reporting the same range of commercial AgNPs to investigate their effects on microalgae. The Ag content of algae filtrates measured by ICP–OES is reported in Figure 2B as percentage of internalised Ag. *C. vulgaris* is able to efficiently take up the AgNPs-G. The Ag content correlates with AgNPs-G amounts used for treatments and with exposure time. Moreover, internalisation of AgNPs increased of about 10% after a week for every treatment, raising the 75% and 86% of internalised NPs at the higher AgNPs-G concentrations (100µg/L and 1 mg/L, respectively). This ability to internalise the AgNPs-G was confirmed by TEM observations (Figure 3(Cd)). AgNPs-G were observed inside large vacuoles or crossing the cell wall (Figure 3(Cd–g)).

EDX microanalysis (Figure 3C) confirms that the electron-dense particles observed inside the microalgae correspond to AgNPs. Interestingly, once inside the microalgae, the NPs are not released back into the medium, either as an active secretion or cell ruptures.

The Ag content of algae filtrates analysed by ICP–OES correlated to the AgNPs-G amounts used for treatments and the time of exposure. The continuous internalisation of AgNPs-G particles observed in our experiments could be dependent on the size and dispersion of our NPs preparation. Data in literature report that bio-adsorption of heavy metal particles to algae is dependent on different properties of NPs, such as surface charge or size, chemical composition, and by the cell walls pore sizes, spanning through the thickness of the walls, ranging from 5 to 20 nm [62,64]. Thus, small nanostructures are highly diffusible, and only NPs up to 20 nm can reach the cell membrane. Other physicochemical properties of AgNPs can influence the internalisation, the rate of entrance and the biological response. Once the cell wall is penetrated, endocytic passage through plasma membrane may be possible and internalised NPs enhance biological effects. Sendra et al. [61] found that the attachment of AgNPs on the surfaces of freshwater and marine microalgae *Chlamydomonas reinhardtii* and *Phaeodactylum tricornutum,* and the presence of AgNPs inside cells directly drives the toxic effects. NPs can also enter into the cells via ion channels, transport proteins and endocytosis mechanism [65].

Also, AgNPs-G enter the algal cells maintaining their crystalline structure once inside even after 1 week. The lack of changes in the crystalline structure of AgNPs was investigated with XRD analysis. Figure 2A shows the XRD pattern of the three Bragg reflections with 2*θ* values of 38.1°, 44.3° and 64.4° which correspond to the (111), (200), and (220) sets of Bragg’s reflections planes of the metallic AgNPs in a sample containing only AgNPs-G and in a sample of *C. vulgaris* treated with AgNPs-G for a week. A sample of only *C. vulgaris* was used as negative control. The spectrum confirmed the face-centred cubic crystalline structure of AgNPs-G with a spherical morphology as characterised by TEM. When AgNPs-G were added to *C. vulgaris* culture, no new diffraction peaks appeared, suggesting that AgNPs-G maintain their crystalline nature. Data in literature report that the microalgae can change the NPs crystalline structure. Studies demonstrated that living *C. vulgaris* showed a capacity to reduce nickel oxide nanoparticles (NiONPs) for zero valence nickel, changing their crystalline structure. The reduction from nanosized NiO to nanosized Ni led to weakened toxicity [40,66]. The maintenance of the crystalline structure of the NPs once inside the microalgae should be analysed as a positive or adverse outcome. In our case the presence of silver in the NPs shape could be a positive aspect as the microalgae can hold silver inside by removing it from the outside environment.

### 3.3. Cell Viability, Chlorophyll Content and Ultrastructure of AgNPs-G Treated C. vulgaris

*C. vulgaris*, at the exponential growth phase, was exposed to Ag ions or AgNPs-G at different concentrations (0.1, 1, 10, 100 μg/L and 1 mg/L) for 1 day and 1 week. The concentration-inhibition graph is reported in Figure 3A. Exposure of algae to AgNPs-G causes a reduction of cell metabolism. The inhibitory rate of growth (IR) increased in a significant way with the increasing time of exposure and doses. In fact, the IR increases up to 6 folds after 1 week of culture in the presence of 1 mg/L of AgNPs-G. Significant growth inhibition was observed in the presence of 100 µg/L and 1 mg/L of AgNPs-G for 24 h. Ag ions exposure induces no effect on cell growth. The negative values of IR at 24 h exposure indicate the so-called hormesis effects of poisoning, both in Ag ions and AgNPs-G treatments.

In line with the growth reduction, the chlorophyll-a concentration reduction (Figure 3B) was dependent on the NPs doses and time of treatment. Statistical analysis revealed a significant difference (*p* < 0.05) between control and treated samples at 24 h in the presence of 1, 10, 100 µg/L and 1 mg/L with a reduction of chlorophyll amount of about 80% at the higher AgNPs-G concentration. Conversely, the treatment of *C. vulgaris* for 1 week induces the decrement in chlorophyll amount in the presence of 10, 100 µg/L and 1 mg/L. The reduction is of about 50% than control cells after culture in the presence of 1 mg/L AgNPs-G. Ag ions exposure induces only a moderate effect on chlorophyll-a content.

TEM ultrastructure of *C. vulgaris* is reported in Figure 3C. In control cells, the plasma membrane was close to the cell wall. Chloroplasts contain well-compartmentalised thylakoids, which are fundamental structures involved in photosynthesis (Figure 3(Ca)). A morphology not different from control cells was observed upon Ag ions treatment with (Figure 3(Cb)). Cells cultured in the presence of the highest AgNPs-G concentration, showed the plasma membrane detaching from the cell wall, (Figure 3(Cc)). The morphological alterations also correlate with AgNPs-G incubation time (Figure 3(Cd)). Large vacuoles with degraded materials were observed (Figure 3(Cd–g), white triangle). A partial structural disorder of thylakoids suggesting a reduced photosynthesis activity is present.

The work reported here confirms *C. vulgaris* an useful microalgae for the detection of the biotoxicity of AgNPs-G, as demonstrated by the fact that culture time and amount of AgNPs influenced growth inhibition, morphological alteration, reduction in chlorophyll-a concentration and photosynthesis perturbation due to structural disorders of thylakoids. The reduced chlorophyll content is in accordance with the data of Hazeem et al., [63] who demonstrated the AgNPs have a negative effect on viability, chlorophyll-a concentration, and increased reactive oxygen species (ROS) formation in *C. vulgaris*. Comparable effects have been demonstrated for other algae species, e.g., *Pithophora oedogonia* and *Chara vulgaris* [27]. Several studies have shown that AgNPs caused inhibition of growth in freshwater green microalgae [67], a reduction in chlorophyll content and morphological changes in the freshwater green alga *Pithophora oedogonia* [27], a decrease of photosynthesis activity in the unicellular green algae *Chlamydomonas reinhardtii* [29], and an increase of antioxidant activities in the marine flagellate *Chattonella marina* [24]. Decrease in chlorophyll content, viable algal cells, increased ROS formation, and lipid peroxidation in the freshwater microalga *Chlorella vulgaris* and marine microalga *Dunaliella tertiolecta* were observed after exposure to AgNPs for 24 h [68].

## 4. Conclusions

In conclusion, it should be kept in mind that the continued increase in the use of AgNPs is a consistent hazard in aquatic ecosystems, where microalgae are key actors, and actions to prevent/reduce this hazard cannot be postponed. Many critical points have to be overcome as the identification of the best biological model for risk assessment, because of species response, exposure conditions and environment-particle chemical interactions. It is thus important to choose the best NPs to be commercialised according to their safety by design synthesis (green chemistry, coatings). In our case, silver nanoparticles are coated with glucose, that ensures stability, in terms of morphology, dispersion and dissolution (release of silver ions). They are stable in several culture media, both in experiments with human cell lines and those with aquatic organisms, as reported in our previous works [42,43]. In BBM culture medium they kept their shape, size and stability, although the medium contained EDTA, which is known to promote the dissolution and dispersion of silver nanoparticles through Ag chelation [69,70]. However, EDTA concentration reported in literature is higher (or not indicated) than concentration used in our experiment, so EDTA effects could be irrelevant on AgNPs-G. Also, it is possible that the presence of the glucose coating interferes in the interaction between EDTA and silver, avoiding the impact of EDTA on the dispersion/dissolution of silver nanoparticles. However, this is a pilot in vitro experiment. Further investigations are needed to understand what can happen on large scale.

Knowledge gaps remain because of the enormous number of nanomaterials (in terms of shape, size, materials coatings, etc.) and the scarce possibility of drawing generalised conclusions.

Microalgal biomass has been applied as a simple and effective alternative to remove heavy metals from aquatic environments. The capacity to adsorb/absorb and accumulate heavy metals in microalgal cells depends on many biotic factors, in particular, the cell density and how algal cells are pretreated before use. The application of suspended algal systems can be limited by the difficulty of removing algae from wastewater after the treatment. The effectiveness of microalgal cells to remove heavy metals can be further enhanced by immobilisation which eliminates the necessity for separating the cells from treated wastewater [71].

Furthermore, the various knowledge gaps are also related to the assessment of functionalised coating toxicity and NPs are still lacking the introduction of a safe approach concept for the use of nanomaterials. Further evaluation is needed to provide suitable methods and procedures to overcome the existing gaps that need to be addressed for the design and production of eco-safe NMs to ensure at the same time marine ecosystem sustainability and remediation.

Our results indicated that exposure to AgNPs-G of *C. vulgaris* caused significant bioaccumulation of nanoparticles and a consequent reduction of microalgae growth and chlorophyll-a content. The internalised NPs were not released back into the medium, even after 1 week, and did not undergo biotransformation since AgNPs-G maintained their crystalline nature. It should be considered that we used an NPs amount that is several times more than the AgNPs released into water. *C. vulgaris* was able to efficiently internalise the AgNPs inside vacuoles and to avoid any volunteer leakages of particles or massive discharge back to the medium for cell disruptions. This bioaccumulation ability of *C. vulgaris* for AgNPs should be taken into consideration for environmental safety and further investigated.
